# Successful resection of port site recurrence of pancreatic ductal adenocarcinoma after laparoscopic distal pancreatectomy

**DOI:** 10.1186/s40792-023-01607-w

**Published:** 2023-03-03

**Authors:** Takashi Aida, Ryota Iwase, Teruyuki Usuba, Yu Kumagai, Kenei Furukawa, Shinji Onda, Masaichi Ogawa, Toru Ikegami

**Affiliations:** 1 Department of Surgery, The Jikei University Katsushika Medical Center, 6-41-2, Aoto, Katsushika-Ku, Tokyo, 125-8506 Japan; 2 Division of Hepatobiliary and Pancreatic Surgery, Department of Surgery, The Jikei University School of Medicine, 3-25-8, Nishi-Shinbashi, Minato-Ku, Tokyo, 105-8461 Japan

**Keywords:** Port site recurrence, Laparoscopic surgery, Pancreatic ductal adenocarcinoma

## Abstract

**Background:**

There are many reports of port site recurrence after laparoscopic surgery for various types of cancer. However, only two cases of port site recurrence after laparoscopic pancreatectomy have been reported to date. We herein report a case of port site recurrence after laparoscopic distal pancreatectomy.

**Case presentation:**

A 73-year-old woman was diagnosed with pancreatic tail cancer and underwent laparoscopic distal pancreatectomy with splenectomy. Histopathological examination revealed pancreatic ductal carcinoma (pT1N0M0 pStage I). The patient was discharged on postoperative day 14 with no complications. However, 5 months after surgery, computed tomography showed a small tumor at the right abdominal wall. No distant metastasis had appeared after 7 months of follow-up. Under the diagnosis of port site recurrence without any other metastases, we resected this abdominal tumor. Histopathological examination showed port site recurrence of pancreatic ductal carcinoma. No recurrence was observed 15 months postoperatively.

**Conclusions:**

This is the report of successful resection of port site recurrence of pancreatic cancer.

## Background

Port site recurrence (PSR) is rarely observed after laparoscopic or thoracoscopic procedures for malignant tumors. Although several reports have described PSR after laparoscopic surgery for colorectal, gallbladder, and gastric cancer, [1–5], only two cases of PSR after laparoscopic pancreatectomy for pancreatic cancer have been published to date [6, 7]. We herein present the case report of PSR after laparoscopic pancreatectomy treated with surgical resection.

## Case presentation

A 73-year-old woman presented to our hospital for evaluation of hematemesis. Her medical history included type 2 diabetes and chronic renal failure, and she was undergoing dialysis.

**Fig. 1:**
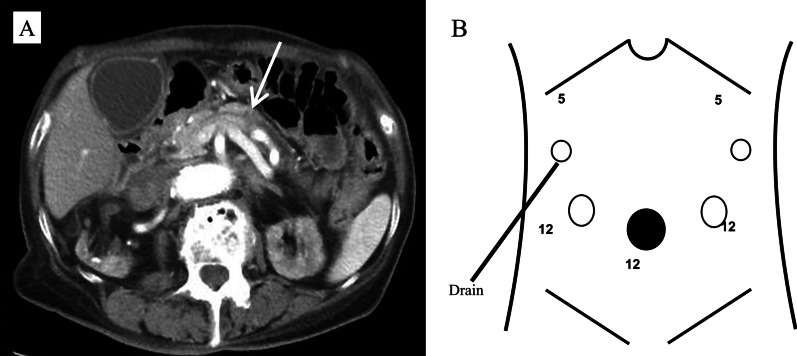
Computed tomography findings and port placement. **A** Computed tomography showed a 12-mm-diameter mass lesion in the pancreatic body with dilatation of the main pancreatic duct (arrow). **B** Two 5-mm ports were placed in the left and right hypochondrium, two 12-mm ports were placed in the left and right lateral abdomen, and one 12-mm port was inserted in the umbilical region by the open method

Upper gastrointestinal endoscopy revealed Mallory-Weiss syndrome. Computed tomography unexpectedly showed a mass lesion in the pancreatic tail with dilatation of the main pancreatic duct (Fig. 1A), and enhanced magnetic resonance imaging also revealed a 12-mm-diameter mass lesion in the pancreatic tail. The serum carcinoembryonic antigen and carbohydrate antigen 19-9 (CA19-9) concentrations were within normal limits (3.8 ng/mL and 26 U/mL, respectively). Endoscopic ultrasound showed a mass lesion in the pancreatic tail, and atypical cells were detected by aspiration cytology using endoscopic retrograde pancreatic drainage. We diagnosed the patient with pancreatic tail carcinoma without lymph node or distant metastasis. Therefore, she underwent laparoscopic distal pancreatectomy with splenectomy. We used two 5-mm ports and three 12-mm ports. Two 5-mm ports were placed in the left and right hypochondrium, two 12-mm ports were placed in the left and right lateral abdomen, and one 12-mm port was inserted in the umbilical region by the open method (Fig. 1B). Carbon dioxide was used for pneumoperitoneum, and the pressure was set to 10 mmHg. The patient was kept in the supine position, and we used a LigaSure™ Maryland jaw device (Medtronic, Minneapolis, MN, USA) for dissection. An Endo GIA™ stapler (Medtronic) was used for transection of the pancreas. Intraoperative cytology was not performed. During surgery, no deviations were observed in any ports and the tumor had not been injured. A drain was placed near the stump of the remnant pancreas through the right 5-mm hypochondrium port site (Fig. 1B).

The operative time was 315 min, and the estimated blood loss was 100 mL. The drain was removed on postoperative day 5, and the patient was discharged on postoperative day 14 without a pancreatic fistula or other complications. The pathological examination revealed moderately differentiated invasive ductal adenocarcinoma and no lymph node metastasis, and curative resection was achieved with 5 mm surgical margins; the pathological stage was IA. Intraductal dilatation of main duct was about 4 mm. The patient did not receive adjuvant chemotherapy because of her renal failure.

**Fig. 2:**
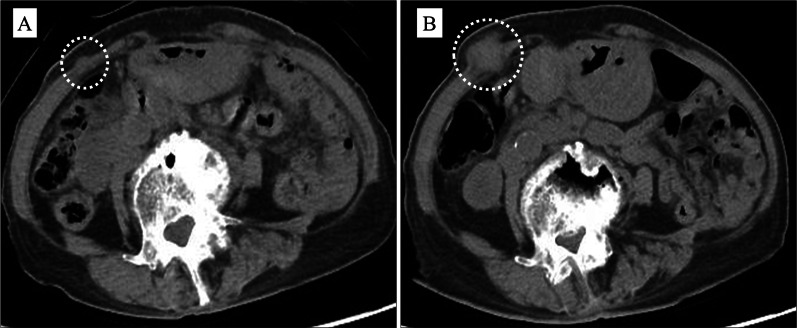
Computed tomography findings after distal pancreatectomy. **A** Five months after surgery, an abdominal wall tumor was found in the 5-mm port site in the right hypochondrium. **B** One year after surgery, the tumor had grown to a diameter of 25 mm. No other intraperitoneal recurrence was detected

**Fig. 3:**
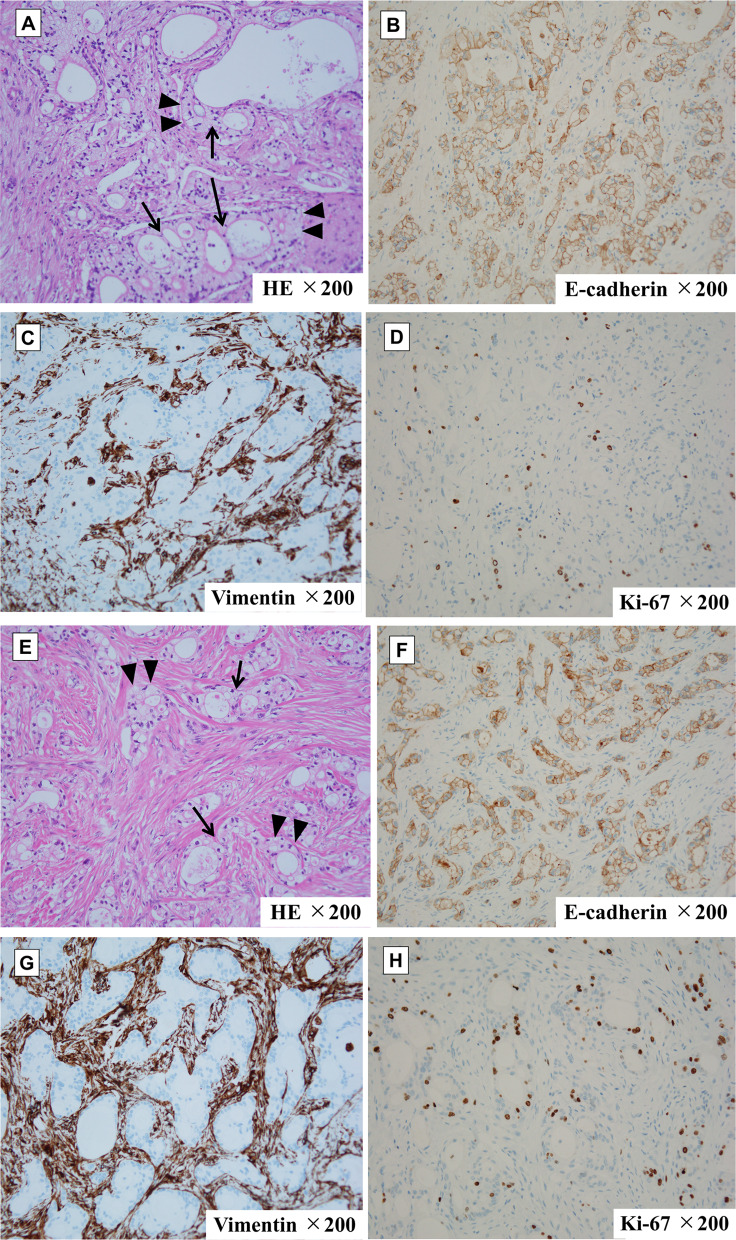
Pathological findings of the resected specimen (magnification × 20). Comparison of **A**–**D** pancreatic specimen from the first surgery and **E**–**H** recurrent specimen from the second surgery. **A**, **E** Hematoxylin- and eosin-stained sections of each specimen revealed ductal adenocarcinoma containing clear cytoplasm (arrowhead) and ductal infusion (arrow). **B**, **F** Immunostaining for E-cadherin showed strong positivity in each adenocarcinoma specimen. **C**, **G** In both specimens, vimentin was expressed only in stromal cells. **D**, **H** Immunostaining for Ki-67 was positive in each adenocarcinoma specimen

Five months after surgery, the patient developed pain and a mass in the right hypochondrium located on the wound of the 5-mm port site, through which the drain had been placed. Her serum carcinoembryonic antigen and CA19-9 concentrations were within normal limits (3.9 ng/mL and 28 U/mL, respectively). Computed tomography demonstrated a 10-mm-diameter mass in the right abdominal wall (Fig. 2A) without any other metastasis. The lesion was considered one of the peritoneal disseminations, or local recurrence of the port site. However, she could not receive any chemotherapy because she had been undergoing dialysis. Therefore, we followed up the patient considering the possibility of peritoneal dissemination without invasive treatment. However, even 10 months after surgery, we could not detect another findings of peritoneal dissemination. Then, we diagnosed the tumor to as the PSR. Because the tumor grew to a diameter of 25 mm and the patient’s CA19-9 concentration increased (111 U/mL) without any other recurrences 10 months later (Fig. 2B), we resected the tumor under general anesthesia. The tumor was 30 mm in diameter, and pathological examination showed well to moderately differentiated adenocarcinoma, similar to the specimen obtained at the time of distal pancreatectomy. In hematoxylin- and eosin-stained sections of each specimen, common features are observed which include clear cytoplasm of ductal carcinoma cell (arrowhead) and back-to-back infusion of each duct (arrow) (Fig. 3A, E). Therefore, we finally diagnosed PSR after laparoscopic distal pancreatectomy for pancreatic cancer. Immunohistochemical examination of each specimen revealed strong positivity for E-cadherin (Fig. 3B, F), negativity for vimentin (Fig. 3C, G), and positivity for Ki-67 (Fig. 3D, F). The patient was discharged on postoperative day 15, and her serum CA19-9 concentration was decreased after surgery. At the time of this writing (15 months after the second surgery), she was clinically well with no evidence of cancer recurrence.

## Discussion

Intra-abdominal surgery has undergone a major shift from laparotomy to laparoscopy. Laparoscopic pancreatectomy has also been introduced for the treatment of pancreatic carcinoma.

The reported incidence of PSR after laparoscopic surgery for various types of cancer is approximately 1%. Though the incidence of PSR after laparoscopic surgery has decreased in recent years, the etiology of PSR remains unclear [2, 3].

Three main mechanisms underlying the cause of PSR have been proposed. The first is that carbon dioxide gas damages the entire peritoneum, promoting the dissemination of tumor cells in the port site [8]. The second is that high-pressure outflow created by pneumoperitoneum leads to the implantation of floating tumor cells in the port wound [2, 9]. The third is contamination of tumor cells by the handling of malignant lesions and bleeding during surgical procedures. However, the correlation between these factors and PSR remains unclear, and how to prevent PSR is not well established. Balli et al. [10] and Emoto et al. [3] focused on prevention of PSR in their reports. Balli et al. [10] indicated that irrigation of the wound and surgical instruments using Betadine (povidone-iodine) might decrease the risk of PSR.

Only two reports of PSR after resection of pancreatic cancer have been published to date [6, 7]. Both patients had pancreatic ductal adenocarcinoma; one was a 49-year-old man who underwent laparoscopic-assisted distal pancreatectomy, and the other was a 57-year-old woman who underwent laparoscopic pancreaticoduodenectomy. Both subsequently received adjuvant chemotherapy and developed PSR with peritoneal dissemination after the therapy (Table 1). In these cases, the PSR might have been one of the peritoneal disseminations. Our patient developed recurrence only at the port site, and no other disseminations were detected.

**Table 1:** Previous reports of PSR after laparoscopic pancreatectomy and present case

Author	Year	Age (years)	Gender	Primary operation	Operation time (min)	Blood loss (ml)	Intraoperative cytology	Stage (UICC 8th edition)	Histology	Adjuvant therapy	Interval of pancreatectomy and PSR (month)	Peritoneal dissemination	Treatment	Outcomes
Koga et al.	2011	49	M	LADP	ND	ND	Negative	III	Mod. adenocarcinoma	GEM	12	Yes	GEM, S-1, Rad	Alive 36 months after primary surgery
Young et al.	2012	57	F	LPD	374	80	ND	IIB	Mod. adenocarcinoma	GEM, Rad	ND	Yes	ND	Died 15 months after primary surgery
Aida et al.	2022	73	F	LDP	315	100	None	IA	Mod. adenocarcinoma	None	5	No	Surgical resection	Alive 27 months after primary surgery

*M* male, *F* female, *LADP* laparoscopic-assisted distal pancreatectomy, *LDP* laparoscopic pancreaticoduodenectomy, *LDP *laparoscopic distal pancreatectomy, *Mod* adenocarcinoma, moderately differentiated adenocarcinoma, *GEM* gemcitabine, *S-1* tegafur, gimeracil and oteracil potassium capsule, *Rad* radiation therapy, *ND* not described

Epithelial–mesenchymal transition (EMT) plays a key role in tumor recurrence and dissemination [11–15]. During EMT, cancer cells lose their adhesive properties and acquire a fibroblast-like morphology and increased motility. During this process, the epithelial biomarker E-cadherin is suppressed and the stromal biomarker vimentin is induced within cancer cells [11, 15]. As the next step in the development of recurrence, these tumor cells regain E-cadherin expression and their epithelial cohesive characteristics while expression of vimentin is downregulated; this is called mesenchymal–epithelial reverting transition [16–18]. In our case, we performed an immunohistochemical examination to investigate whether EMT had been induced in the recurrent cancer tissue. However, the expression of E-cadherin in the recurrent tissue was positive and that of vimentin was negative, suggesting that EMT was not present in the recurrent cancer tissue. In Ki-67 staining, the MIB-1 index of the recurrent cancer tissue was higher than that of the primary pancreatic cancer (13.2 and 6.7, respectively). This may reflect more aggressive behavior of the recurrent tumor than of the primary tumor. More comprehensive research like microarray analysis is needed to further investigate EMT in such cases.

The PSR in this case was present on the abdominal wall at the site of the surgical drain, and the pancreatic carcinoma cells of the PSR might have been delivered via the surgical drain at the time of its removal. Actually, a few reports have described drain site metastasis after laparotomy in recent years [19, 20]. In the two reports of PSR of pancreatic cancer, however, the location of the drain site was not described. Therefore, whether the recurrence occurred at the drain site is unknown.

Why the PSR was occurred, despite the pathological staging was IA, is unclear. As we mentioned previously, it might be possible that a few tumor cells which had been in pancreatic juice intra remnant pancreas, leaked out and adhered to the drain after surgery. During removal of the drain, tumor cells might attach to the wound site, and it had grown into PSR. Moreover, circulating tumor cell (CTC) is collecting a lot of attention as a biomarker for early recurrence or prognosis. According to the reports, in even early pancreatic cancer including Stage I, CTC was detected from portal blood or central venous, and CTC presence in portal vein is related to poor prognosis [21, 22]. Therefore, even in our patient, CTC might have existed in the blood after the pancreatectomy, and it may be possible that CTC affected the PSR.

According to previous reports, the main therapeutic strategy of PSR may be chemotherapy, radiotherapy, or surgical resection [23, 24]. Several reports have described successful local resection of PSR that had occurred without peritoneal dissemination and/or distant metastasis. These patients achieved long-term survival after PSR resection [4, 23, 26]. Wang et al. [27] suggested that surgical resection is an effective treatment for PSR without another peritoneal dissemination. In the present case, we followed up the patient for 6 months after she became aware of the abdominal mass lesion. Because no evidence of peritoneal dissemination or distant metastasis was detected throughout this observation period, we performed surgical resection of the PSR. The patient was free of recurrence 1 year after the second surgery, and surgical resection might have therefore been a curative treatment of PSR for this patient. With the increase in performance of laparoscopic pancreatectomy, the occurrence of PSR after resection of pancreatic carcinoma is also expected to increase.

## Conclusions

We have herein reported a case of PSR after laparoscopic pancreatic surgery for pancreatic cancer with successful resection of the recurrent tumor. Radical resection of PSR of pancreatic cancer is a possible treatment if the patient has no evidence of peritoneal dissemination at another site.

## Data Availability

The data that support the findings of this study are available from the corresponding author upon reasonable request.

## Abbreviations

PSR: Port site recurrence
CA19-9: Carbohydrate antigen 19-9
EMT: Epithelial–mesenchymal transition
CTC: Circulating tumor cell
